# Repurposing Probenecid for the Treatment of Heart Failure (Re-Prosper-HF): a study protocol for a randomized placebo-controlled clinical trial

**DOI:** 10.1186/s13063-022-06214-y

**Published:** 2022-04-07

**Authors:** Jack Rubinstein, Nathan Robbins, Karen Evans, Gabrielle Foster, Kevin Mcconeghy, Toluwalope Onadeko, Julie Bunke, Melanie Parent, Xi Luo, Jacob Joseph, Wen-Chih Wu

**Affiliations:** 1 Division of Cardiovascular Medicine, Cincinnati Veterans Affairs Medical Center, 3200 Vine St, Cincinnati, OH 45220 USA; 2 Department of Internal Medicine, Division of Cardiovascular Diseases, College of Medicine, University of Cincinnati Medical Center, Cincinnati, OH USA; 3 Ohio University, Heritage College of Osteopathic Medicine, Athens, OH USA; 4 Medical Service and Center of Innovation for Long Term Services & Support, Providence Veterans Affairs Medical Center, Providence, USA; 5 Massachusetts Veterans Epidemiology Research and Information Center and Medical Service, VA Boston Healthcare System, Boston, MA USA; 6 Providence Veterans Affairs Medical Center, Providence, USA; 7 Veterans Affairs Medical Center, Cincinnati, OH USA; 8 Department of Research, Cincinnati Veterans Affairs Medical Center, Cincinnati, OH USA; 9 Center of Innovation for Long Term Services & Support, Providence Veterans Affairs Medical Centers, Providence, USA; 10 Department of Biostatistics and Data Science, School of Public Health, The University of Texas Health Science Center at Houston, Houston, USA; 11 Cardiology Section, VA Boston Healthcare System, Boston, MA USA; 12 Division of Cardiovascular Medicine, Department of Medicine, Brigham & Women’s Hospital, Boston, MA USA; 13 Medical Service and Center of Innovation for Long Term Services & Support, Providence Veterans Affairs Medical Center, Providence, USA; 14 Department of Medicine, Alpert Medical School, Providence, USA; 15 Department of Epidemiology, Brown University, Providence, USA

**Keywords:** Probenecid, Heart failure, Contractility, Inotropy

## Abstract

**Background:**

Improving contractility in heart failure with reduced ejection fraction (HFrEF) has resurfaced as a potential treatment goal. Inotropic therapy is now better understood through its underlying mechanism as opposed to the observed effect of increasing contractility. Calcitropes are a subgroup of inotropes that largely depend on the stimulation of adenylyl cyclase to transform ATP into cyclic adenosine monophosphate (cAMP). At least two clinically relevant calcitropes—istaroxime and probenecid—improve contractility through an increase in systolic intracellular calcium without activating cAMP production.

Probenecid, which has been safely used clinically for decades in non-cardiac conditions, has recently been identified as an agonist of the transient receptor potential vanilloid 2 channel. Translational studies have shown that it improves calcium cycling and contractility without activating noxious pathways associated with cAMP-dependent calcitropes and can improve cardiac function in patients with HFrEF.

**Methods:**

The Re-Prosper-HF study (Repurposing Probenecid for the Treatment of Heart Failure with Reduced Ejection Fraction) is a three-site double-blinded randomized-controlled trial that will test the hypothesis that probenecid can improve cardiac function in patients with HFrEF. Up to 120 patients will be randomized in this double-blind, placebo-controlled study that will assess whether oral probenecid administered at 1 g orally twice per day for 180 days in patients with NYHA II-III HFrEF improves systolic function (aim 1), functional status (aim 2), and self-reported health status (aim 3).

**Discussion:**

Findings from this study will provide data informing its use for improving symptomatology in patients with HFrEF as well as exploratory data for outcomes such as hospital admission rates.

**Trial tegistration:**

The Re-Prosper HF Study (Re-Prosper HF) is registered on ClinicalTrials.gov with the identifier as NCT04551222. Registered on 9 September 2020.

## Administrative information

Note: the numbers in curly brackets in this protocol refer to SPIRIT checklist item numbers. The order of the items has been modified to group similar items (see http://www.equator-network.org/reporting-guidelines/spirit-2013-statement-defining-standard-protocol-items-for-clinical-trials/).

**Table:** 

Title {1}	Repurposing Probenecid for the Treatment of Heart Failure (Re-Prosper-HF): a study protocol for a randomized placebo-controlled clinical trial
Trial registration {2a and 2b}.	The Re-Prosper HF Study (Re-Prosper HF) is registered on ClinicalTrials.gov with the identifier as NCT04551222.
Protocol version {3}	This is version 1.0 of the protocol date September 4, 2020
Funding {4}	This study was funded by grant number VA-CSR&D I01 CX001968 through the Veterans Affairs.
Author details {5a}	Jack Rubinstein, MD, Division of Cardiovascular Medicine, Cincinnati Veterans Affairs Medical Center and Department of Internal Medicine, Division of Cardiovascular Diseases, College of Medicine, University of Cincinnati Medical Center, Cincinnati Ohio, Jack.rubinstein@va.gov Nathan Robbins, MS, Ohio University, Heritage College of Osteopathic Medicine, Athens, Ohio, robbins.nj@gmail.com Karen Evans, RN, Medical Service and Center of Innovation for Long Term Services & Support, Providence Veterans Affairs Medical Center, karen.evans7@va.gov Gabrielle Foster, Massachusetts Veterans Epidemiology Research and Information Center and Medical Service, VA Boston Healthcare System, Boston, MA, Gabrielle.Foster@va.gov Kevin Mcconeghy, PharmD, MS, BCPS, Research Service, Providence Veterans Affairs Medical Center, Kevin.Mcconeghy@va.gov Toluwalope Onadeko, PharmD, MBA, MS, BCGP RPH, Pharmacy Service, Cincinnati Veterans Affairs Medical Center, Cincinnati, Ohio, Toluwalope.Onadeko@va.gov Julie Bunke, BA, CCRP, Department of Research, Cincinnati Veterans Affairs Medical Center, Cincinnati, Ohio, Julie.bunke2@va.gov Melanie Parent, BA, Research Service, Center of Innovation for Long Term Services & Support, Providence Veterans Affairs Medical Centers, Melanie.Parent@va.gov Xi Luo, PhD, The University of Texas Health Science Center at Houston, School of Public Health, Department of Biostatistics and Data Science, rossi.stat@gmail.com Jacob Joseph, MBBS, MD, Cardiology Section, VA Boston Healthcare System, Boston, MA and Division of Cardiovascular Medicine, Department of Medicine, Brigham & Women’s Hospital, Boston, MA, Jacob.Joseph@va.gov Wen-Chih Wu, MD, MPH, Medical Service and Center of Innovation for Long Term Services & Support, Providence Veterans Affairs Medical Center and Department of Medicine, Alpert Medical School & Department of Epidemiology, Brown University, Wen-Chih.Wu@va.gov
Name and contact information for the trial sponsor {5b}	This trial is investigator initiated and funded through the grant applications. There is no industry sponsor.
Role of sponsor {5c}	This trial is investigator initiated and funded through the grant applications at the Office of Research & Development at the Department of Veterans Affairs. There is no industry sponsor. The funders reviewed previous versions of the proposal and submitted suggestions which were incorporated into the final funded protocol. There is an independent Data Safety Management Board as well as an Institutional Review Board to monitor the study, though these will not have authority over the writing of the report nor the decision to submit it for publication.

## Introduction

### Background and rationale {6a}

Heart failure with reduced ejection fraction (HFrEF) affects millions of people worldwide. The prevalence increases with advancing age and despite therapeutic advances over the last few decades, there is still a dismal prognosis (mortality of about 75% at 5 years) [1]. Heart failure (HF) has been the number one reason for admission among patients in the Veterans Affairs Health Care System [2], and even with improved therapeutic options, the hospitalization rate has remained stable while rehospitalization rates may have increased [2].

**Fig. 1:**
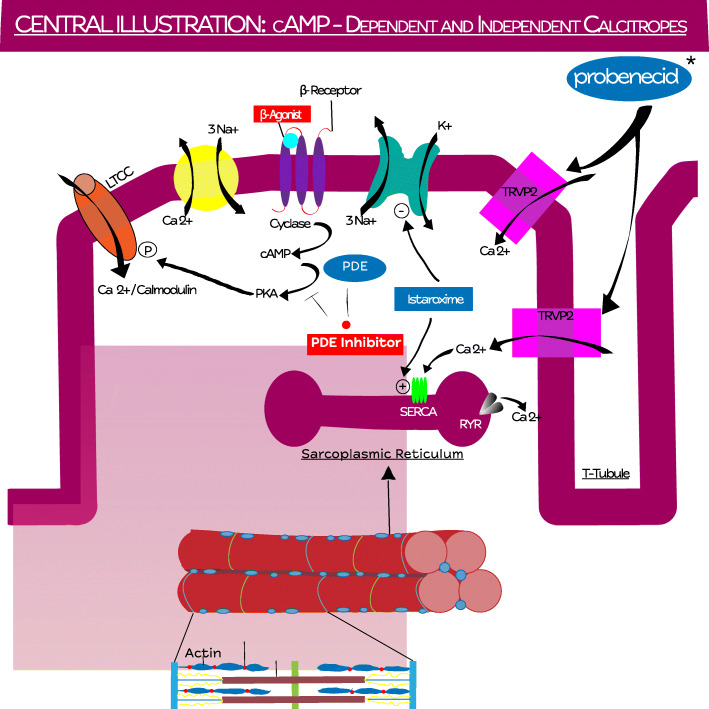
Central illustration: cAMP-dependent and -independent calcitropes. Summary figure demonstrating mechanism of action of current medical therapies for the treatment of heart failure with reduced ejection fraction. LTCC, L-type calcium channel; Ca2+, calcium; NA+, sodium; cAMP, cyclic adenosine monophate; PKA, protein kinase A; PDE, phosphodiesterase E; K+, potassium; TRPV2, transient receptor potential vanilloid 2; SERCA, sarcoendoplasmic reticulum calcium transport ATPase; RYR, ryanodine receptor

Inotropic options for treatment of HFrEF have recently been categorized through their mechanistic underpinnings as calcitropes, myotropes, and mitotropes [3]. This classification distinguishes between traditional cAMP-related calcitropic inotropes and novel inotropes, such as those with effects on myofilaments (myotropes) or those altering metabolism at the level of the mitochondria (mitotropes). Calcitropes possess a shared pathway of increasing calcium flux into the myocyte; however, there are differences in the mechanisms by which calcitopes affect calcium flux, which has clinical implications. The traditional inotropes (i.e., dobutamine, ibopamine, dopamine, and xamoterol) accomplish this through beta-adrenergic and downstream stimulation of adenylyl cyclase to transform ATP into cyclic adenosine monophosphate (cAMP) or through inhibition of phosphodiesterase-3 with similar downstream effects (i.e., milrinone, enoximone, vesnarinone, pimobendan, and levosimendan) [4]. There is a subgroup of calcitropes, described herein as non-cAMP calcitropes that are able to increase calcium flux within the cardiomyocyte without cAMP activation or its reported adverse effects (arrhythmias, apoptosis, hypertrophy). The two compounds that have reached advanced clinical studies are istaroxime, an inhibitor of the sarcolemmal Na^+^/K^+^ pump and activator of the SERCA2a pump, and probenecid, an agonist of transient receptor potential vanilloid 2 (TRPV2) channels that has been shown to increase calcium influx and calcium-induced calcium release (CICR). Where these compounds interact with the cardiomyocyte can be seen in Fig. 1.

The distinction between cAMP and non-cAMP calcitropes is crucial as the former have been well-documented to increase mortality in patients with HFrEF [5]. These studies have examined thousands of patients at various stages of disease progression and under several clinical conditions, most if not all of which, have reported worsening outcomes associated with their use. The main culprits of increased mortality are proposed to be their vasoactive effects (both dilation and constriction) as well as increasing heart rate and risk of arrhythmias. These findings are in stark contrast with clinical studies using non-cAMP calcitropes such as istaroxime, which has shown only slight changes in blood pressure and heart rate at low doses (more pronounced at higher doses), while probenecid has shown no significant vasoactive, chronotropic, or arrhythmogenic effects [6].

#### Probenecid: mechanism of action and preclinical studies

Probenecid was initially described as an agonist of the TRPV2 channels in hamster ovaries overexpressing the channel [7]. Subsequently, it was described as an agonist of TRPV2 channels in cardiomyocytes with potent inotropic effects in vitro and in several in vivo animal models [8]. The in vitro studies demonstrated that probenecid-induced agonism of TRPV2 increased intracellular calcium flux with increased CICR resulting in augmented intracellular calcium handling, particularly during diastole [9]. Furthermore, we have observed that TRPV2 is upregulated in diseased hearts in which we observed more profound stimulation of TRPV2-mediated Ca^2+^ influx, greater augmentation of SERCA activity, and increased Ca^2+^ release and contractility [9].

Concurrent animal models, both healthy and diseased, confirmed the in vitro observations of increased contractility. Specifically, murine ischemic models demonstrated dramatically increased contractility after treatment with probenecid that was inversely proportional to the degree of baseline dysfunction, consistent with higher expression of myocardial TRPV2 under stress conditions [9]. Other studies, including a murine model of peripartum cardiomyopathy and in vivo porcine model documented similar increases in contractility without increased arrhythmias or mortality [10, 11].

#### Clinical studies

The potential clinical benefit of probenecid in HFrEF was described by our group in a pilot phase 2 study that tested probenecid at 1 gr. orally twice daily (as proposed in this study) for at least 3 months in 20 adult patients with previously established compensated HFrEF (LVEF≤40%), in a randomized, double-blind, cross-over, placebo-controlled trial. On treatment with probenecid, systolic and diastolic function improved in comparison to placebo without adverse effects [12]. A second study on young subjects with hypoplastic left heart syndrome treated with Fontan’s procedure also showed improved systolic function without adverse effects [13].

More recently, a retrospective study of nearly 40,000 Medicare recipients compared outcomes in subjects who had initiated probenecid in comparison to allopurinol for hyperuricemia and found that the incidence of hospitalization for HF exacerbation in patients with baseline HF was significantly lower in the probenecid group. Although the study cannot elucidate the drug effect by LVEF, it provides indirect evidence in a large population that probenecid is safe and may improve outcomes in HF [14].

### Objectives {7}

Based on the pre-clinical, clinical, and population-based data we are conducting this study to evaluate the effects of probenecid in a stable HFrEF population in a prospective, randomized-controlled study design, to test the hypothesis that probenecid will improve cardiac function in patients with HFrEF. The primary outcome of the trial is improved cardiac function after 6 months as measured by the change in ejection fraction on echocardiography. Additional outcomes include exercise tolerance and symptomatology using standardized validated questionnaires.

### Trial Design {8}

The Re-Prosper-HF Trial is a three-site, double-blinded, randomized, placebo-controlled, parallel design, trial in a 1:1 fashion of 1 gr. orally of probenecid twice daily or identical placebo for 180 days. We propose to recruit 120 subjects (power analysis below) with HFrEF (LVEF≤40%), NYHA II-III, on guideline-directed medical therapy.

## Methods: Participants, interventions, and outcomes

### Study setting {9}

The study will take place at the Veterans Affairs Medical Centers in Cincinnati, Providence, and Boston. The protocol was approved by the ethics committees and Institutional Review Boards prior to the initiation of any study procedures. The trial is registered on ClinicalTrials.gov (NCT04551222).

### Eligibility criteria {10}

The inclusion and exclusion criteria as outlined in Table 1 will be used to enroll patients with stable heart failure on guideline direct medical therapy. Briefly, patients must have a documented history of systolic dysfunction within the last 12 months and be on a current medical regime for systolic heart failure for at least 2 weeks without any new medications or dose changes [15]. Patients with a recent myocardial infarction, revascularization, or chronic resynchronization therapy will be temporarily excluded. Additional exclusion criteria have been used to ensure patient safety including renal failure with eGFR < 30 ml/min (since probenecid is primarily excreted by the kidneys) and medications that interact with probenecid [16].

**Table 1:** Selected inclusion and exclusion criteria for the Re-Prosper-HF Trial

Key inclusion criteria	Key exclusion criteria
Have documented heart failure as a treated inpatient or outpatient diagnosis in the medical record. Left ventricular ejection ≤40% within the past 12 months either by echocardiogram, cardiac MRI, cardiac CT, nuclear imaging or cardiac catheterization. NYHA class II–III On stable Guideline-directed medical therapy for at least 2 weeks (including at least an EBM dose of betablocker and RAAS inhibition) consistent with the EPHESUS trial criteria or having a documented allergy or adverse reaction to betablocker and/or RAAS inhibition. Age 18 years or older.	Acute coronary syndrome or cardiac revascularization within the past 3 months. End-stage renal disease with renal replacement therapy or creatinine clearance less than 30 ml/min. Cardiac resynchronization therapy within the past 3 months. Constrictive pericarditis or restrictive cardiomyopathy on cardiac imaging study (echocardiogram cardiac MRI, cardiac CT) within the last 12 months. Ablation for cardiac arrhythmias within the past month. Peripartum cardiomyopathy diagnosed within past 6 months. If LVEF is still ≤40% after 6 months of diagnosis, they can be enrolled into the study. Uncorrected cyanotic congenital heart disease. Greater than moderate degree of stenotic or regurgitant valvular disease. Women who are pregnant, breast feeding, or plan to become pregnant during the study. All women in childbearing age will undergo baseline and quarterly urine pregnancy tests to ensure absence of pregnancy since the cardiometabolic assessments will be different during pregnancy. Terminal illness with expected survival of less than 12 months. Oral therapy with probenecid for any indication during the preceding 3 months. Hypersensitivity to probenecid based on prior exposure. Inability to provide informed consent or study procedures due to dementia, unstable psychiatric disease, or other cause (e.g., inability to do perform exercise testing). Acute gout attack within the previous 3 months. History of uric acid kidney stones within the last year. Patient will be removed from the study if they develop urate kidney stones. History of blood diseases in the past year: Aplastic anemia, Hemolytic anemia, Leukopenia, Neutropenia, Pancytopenia, Thrombocytopenia or leukemia. Creatinine clearance (eGFR) < 30 ml/min. *Patients on the following medications that has potential interaction with probenecid will need to be reviewed by study PI prior to enrollment: cephalosporins, quinolones, penicillins, methotrexate, zidovudine, ganciclovir, and acyclovir; since the excretion of these drugs is reduced due to probenecid. If a patient is started on any of these medications the physician will be advised that it may increase their serum levels.

### Who will take informed consent? {26a}

Informed consent will be obtained by study staff designated by the principal investigator at each site. Potential participants including pre-screened inpatient or outpatient will be contacted using one of the approved follow-up methods and is agreeable to come in for the initial study visit. Once the consent has been reviewed with the patient and all questions answered, we will consent and enroll the patient into the study.

### Additional consent provisions for collection and use of participant data and biological specimens {26b}

Not applicable.

## Interventions

### Explanation for the choice of comparators {6b}

The dose of probenecid used in this trial (1 g twice a day) has been chosen based on preclinical and previous clinical trials and is the dose that was initially FDA approved for use in the treatment of gout. Additionally, this is the dose that was used in the pilot, phase II clinical trial that was proven to be safe and effective in patients with heart failure. Matching placebo will be used as the control and instructed to be taken identically, twice a day for 6 months.

### Intervention description {11a}

**Fig. 2:**
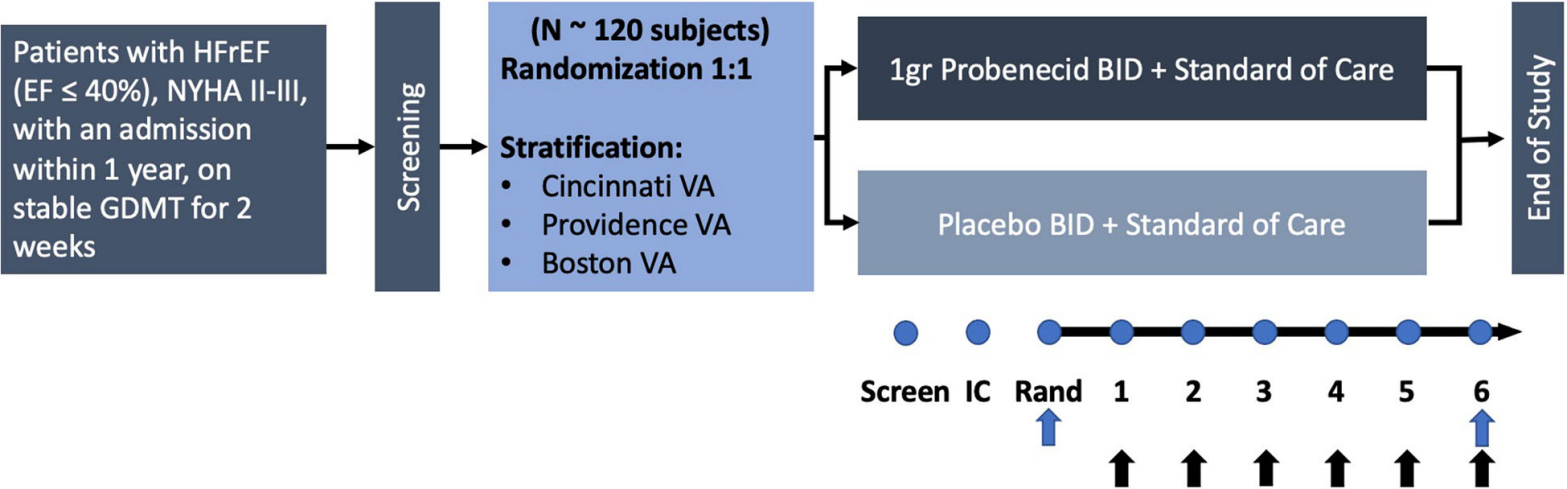
Schematic of study design for the Re-Prosper-HF Trial. Study flow diagram summarizing major inclusion criteria, screening, randomization, stratification by site, and study visits. HFrEF, heart failure with reduced ejection fraction; EF, ejection fraction; GDMT, guideline-directed medical therapy; VA, Veterans Affairs Hospital; BID, twice daily; Screen, screening visit; IC, informed consent; Rand, randomization

Patients will be randomized in a 1:1 fashion via block randomization of size 4, stratified by study sites for patient assignments. The study will be blinded to all participants, investigators, and other study personnel except the Pharmacy team. The active study drug, probenecid, and the placebo are identical visually (Fig. 2).

### Criteria for discontinuing or modifying allocated interventions {11b}

Adverse events determined by study staff will be reported to the principal investigator of the site where the event occurred. The investigator will determine whether or not the event is clinically likely to be due to probenecid and will determine whether or not to discontinue the study medication. This event will be reported to the Data Safety Monitoring Board and the Central Institutional Review Board who will review the event and determine whether it is safe to continue the study. There are currently no plans to decrease the dose of probenecid as the dose used has proven clinically safe over decades of use.

### Strategies to improve adherence to interventions {11c}

A pill count will occur during interval visits to assure patients are taking study medications properly. If it is determined that the participants are not taking medications properly, they will be educated and the study staff will make note of this occurrence as a note to file.

### Relevant concomitant care permitted or prohibited during the trial {11d}

Not applicable.

### Provisions for post-trial care {30}

Participants will receive a phone call after the study to ensure safety after completion of the trial.

### Outcomes {12}

#### Primary outcome

Change from baseline in cardiac function (Specific Aim 1). The main primary outcome is echocardiogram-derived EF. We will use Definity® echo contrast if adequate endocardial border definition cannot be ascertained for ejection fraction calculation in apical 4 and apical 2 using volumetric tracing analysis and modified Simpson’s. The echosonographers from all sites will follow the same standard study procedure to obtain the views. Briefly, standard B-mode images will be obtained from the apical and parasternal views for left ventricular chamber size and function. We will also measure function via global longitudinal strain, using a reproducible and objective measure by TomTec® software AutoStrain© function using speckle tracking of apical long-axis views. All studies will be read centrally by a single, blinded, board-certified echocardiographer using the standards as set forth by the 2015 American Society of Echocardiography Guidelines [17].

#### Secondary outcome

Change from baseline in exercise tolerance via a symptom-limited exercise test on a cycle ergometer (Specific Aim 2). Most patients with HFrEF report reduced functional status and impaired quality of life. Thus, the importance of improving (and measuring) symptoms and functional status cannot be overemphasized. We will perform a maximal bicycle exercise stress test (BEST) on a cycle ergometer with 10-W workload increments at 1-min intervals [18] to adapt to patients with low functional capacity as previously described and with all the standard precautions.

At the end of the test, the cycle ergometer will provide the total workload in Watts (W), which will be converted to Metabolic Equivalents (METS) using the standard American College of Sports Medicine formula for leg ergonometry (American College of Sports Medicine. ACSM’s Guidelines for Exercise Testing and Prescription): METS* = [(1.8*W*6/weight in kg of the subject) + 7]/3.5 formula and compared to baseline [19]. Since the purpose of the test is to obtain a measure of the maximal functional capacity as opposed to maximal heart rate response, patients will be instructed to continue on all their cardiac medications, specifically their beta-blockers and ACE-Inhibitors or angiotensin receptor blockers

#### Tertiary outcome

Change from baseline in The Kansas City Cardiomyopathy Questionnaire (KCCQ) and EQ5D (Specific Aim 3). The KCCQ & EQ5D will be completed before any procedures or tests (if needed will provide the appropriate translated versions or will be read to the patients by the staff). The KCCQ is a 23-item instrument validated in stable and decompensated HF patients with HFpEF and HFrEF. The domains of KCCQ are physical limitation, symptoms, self-efficacy, social limitation, and quality of life. It has a significantly higher responsiveness to changes in health status than the Minnesota Living with Heart Failure Questionnaire and the SF-36 for HF [20].

Overall health status (EQ5D) is a well-known generic measure of health status. It has 5 questions that address five dimensions (mobility, self-care, usual activities, pain/discomfort, anxiety, or depression) with five levels ranging from ‘no problems’ to ‘extreme problems’ each [21]. The EQ5D has moderate-strong convergent validity in relation to other measures of health-related quality of life as well as discriminative validity to detect clinical changes.

##### Laboratory values, echocardiograms, and routine assessments

Baseline and safety laboratory studis to be collected include liver and kidney panels as well as a complete blood count to assess for anemia or infection. Cardiovascular disease laboratory studies will also be collected including B-type natriuretic peptide, troponin, a twelve-lead electrocardiogram, and a complete echocardiographic evaluation as described above. Patients will perform a maximal BEST on a cycle ergometer with 10-W workload increments at 1-min intervals [18] to adapt to patients with low functional capacity as previously described and with all the standard precautions. The patients will complete the KCCQ as well as EQ5D. The echocardiographic study, BEST, and questionaries will be performed at the first visit and again at the final visit to assess if the investigational product (IP) had any effect on the parameters measured.

### Participant timeline {13}

After patients have been randomized and baseline data will be collected including echocardiogram, exercise tolerance test, questionnaires, electrocardiogram, and laboratory studies. Study outcomes will be assessed at baseline (prior to the start of study medication) and at 6 months. Safety measures will be assessed at monthly visits. Once baseline data has been collected, they will be sent home with IP and instructed to take pills twice daily. Patients will be asked to return every month for 6 months for assessing safety and adherence to the IP. Data collected will include safety laboratory studies, electrocardiogram, and any reported changes in medications. Hospitalizations or adverse events will be noted and will be reported to all regulatory authorities. On the final visit, study outcomes will be measured including echocardiography, exercise tolerance test, and questionnaires as well as final safety laboratory studies and electrocardiogram. Patients will receive a phone call after the study to ensure safety after the IP has been stopped.

### Sample size {14}

Sample size estimations were calculated using t-tests based on a pilot study [12]. All sample calculations were carried out using SAS 9.4. For the primary outcome, based on data from the pilot study, a sample size of 60 in each group will have 99% power to detect a difference in means of 7.67 (the difference between a Group 1 mean, μ_1_, of 4.67 and a Group 2 mean, μ_2_, of − 3) assuming that the common standard deviation is 9.72 using a two-group *t* test with a 5% two-sided significance level. Even after accounting for a worse-case attrition rate of 20%, the complete case analysis of 48 subjects in each group will retain 96.9% power.

For exercise tolerance, 6-min walk test data from the pilot study was converted to METS using the ACSM conversion table to estimate the power for the outcome, which is METS measured by exercise bike test. Six-minute walk test improved 21.1 ± 24.9 feet (equivalent to 1.03 ± 1.04 METS) in the probenecid arm vs −22.7 ± 24.9 feet (equivalent to − 1.03 ± 1.04 METS) in the placebo arm. So, a sample size of 60 in each group will have > 99% power to detect a difference in means of 2.06 (the difference between a Group 1 mean, μ_1_, of 1.03 and a Group 2 mean, μ_2_, of − 1.03) assuming that the common standard deviation is 1.04 using a two-group *t* test with a 5% two-sided significance level. The power remains > 99% after accounting for the worst-case attrition rate of 20%.

### Recruitment {15}

Inpatient floors and outpatient clinics will be screened for patients admitted for heart failure or with a history of heart failure noted in their chart. Reduced ejection fraction will be verified by reviewing reports of imaging studies including echocardiography, cardiac magnetic resonance imaging, computerized tomography, nuclear imaging, or cardiac catheterization. Potential subjects will be approached with the approval of their treatment team or providers. If the patient verbally agrees, a screening visit will be scheduled.

## Assignment of interventions: allocation

### Sequence generation {16a}

Randomization will occur at each site and will be performed by the pharmacist or designated staff in a 1:1 fashion via block randomization of size 4. Pharmacy staff will maintain records regarding group designation. If necessary, the Data Safety Monitoring Committee may unblind themselves if at any time patient safety is a concern.

### Concealment mechanism {16b}

Investigation product (IP) will be dispensed in identical capsules whether probenecid or placebo. Only the central statistician will have access to records in regards to group allocation.

### Implementation {16c}

Research staff enrolling the participant will contact pharmacy staff to prepare the (IP). Pharmacy will distribute the IP according to the randomization table and record group allocation. Research staff will retrieve IP and dispense to the participant with dosing instructions.

## Assignment of interventions: blinding

### Who will be blinded {17a}

Besides pharmacy, all study staff and participants will be blinded to medication allocation.

### Procedure for unblinding if needed {17b}

If participant safety is a concern and the principal investigator deems the event likely due to IP, the participant will stop taking the product. If necessary, the appropriate care team can be unblinded to the study group.

## Data collection and management

### Plans for assessment and collection of outcomes {18a}

Data will be collected at appropriate visits on case report forms and entered into an electronic database. Blank case report forms will be stored at the individual sites in secure designated research offices. This database will be queried periodically during the study and after all patients have been enrolled to verify data entered is appropriate and within standard ranges. If inappropriate data is discovered or data is outside of the physiologic range, study staff will be notified to remedy the error. Data collection forms are located in the study protocol for study staff, or the forms can be obtained by contacting the corresponding author.

### Plans to promote participant retention and complete follow-up {18b}

Participants will be followed up on regular intervals. Pill counts will be completed to ensure proper administration of the IP. Participants may receive a phone call to remind them of their upcoming research follow-up appointment. For patients who discontinue, no study outcome data can be collected given that they require active patient participation (echocardiography, bike exercise test, questionnaires). Protocol non-adherence will be noted. If there is a significant protocol violation that compromises the scientific rigor as determined by the data safety monitoring board, data will not be included in the analysis per recommendations of the data safety management board. If data is missing, it will not be included in the analysis.

### Data management {19}

All information collected at each visit are recorded on case report forms and entered electronically by study staff into an electronic database REDCap®. Paper forms will be maintained at each site and scanned electronically. Electronic data will be stored in a study shared folder that is encrypted and under password protection. Data quality will be audited on a periodical basis for accuracy for every participant by a blinded database manager and the database will be locked at the end of the trial to allow the statistician blinded to the study allocation to complete the analysis.

### Confidentiality {27}

All efforts will be made to maintain participant confidentiality during the study. Each participant will be assigned a study identification and that will be used on case report forms and electronic data. The participant’s name and study identification will be on the enrollment log and will only be accessed by study staff.

### Plans for collection, laboratory evaluation, and storage of biological specimens for genetic or molecular analysis in this trial/future use {33}

Not applicable.

## Statistical methods

### Statistical methods for primary and secondary outcomes {20a}

The primary analysis will be performed from an intention to treat principle, and the secondary analysis will include as-treated analysis and sensitivity assessment. Descriptive statistics (such as mean, standard deviation, median, inter-quartile range, and frequency distribution), as appropriate to each variable’s distribution, will be calculated to summarize participants’ baseline demographic and medical history, medication, and functional status information including medication list (specifically, ACEI, or ARB or ARNI and beta-blocker use) and anthropometric measures, such as blood pressure and the study outcomes according to randomized intervention sequences. We will compare baseline characteristics to assess whether a balanced randomization is accomplished at baseline using t or nonparametric (Wilcoxon’s) tests, and chi-square tests whenever each test’s distribution assumption is appropriate. Log-transformation of variables and outcomes of interest will be performed to approximate normality when appropriate. We will also use these statistics to assess if there are site differences or site-treatment interactions using the tests appropriate for the distributions.

For the intention-to-treat analysis, we will use t statistics and normal approximation to assess the significance of the randomization effects. To adjust for potential imbalance of baseline characteristics, linear regression modeling with sites as a strata factor will be utilized, where the response variable is the outcome measured, the main effect will be randomization (probenecid or placebo), and covariates will be baseline outcome and imbalanced covariates at baseline identified in the descriptive analyses between the study arms. Residual diagnostic and influence analysis will be conducted to examine model fitting, assumptions, and assess outlier observations. As a secondary analysis for assessing compliance, as-treated analysis (accounting for pill count results) will also be carried out using the same modeling approach.

### Interim analyses {21b}

We will perform a blinded interim analysis at 50% of the recruitment target. The study will proceed as planned unless the difference in the absolute rate of adverse events between the two study arms are greater 20%, at which case the study will be halted and a balance of the risk vs benefit ratio of completing the study will be discussed with the Data Safety Monitory Board. We will perform blinded sample size re-estimation [22] during our interim analysis.

### Methods for additional analyses (e.g., subgroup analyses) {20b}

Not applicable.

### Methods in analysis to handle protocol non-adherence and any statistical methods to handle missing data {20c}

Protocol non-adherence will be noted. If there is a significant protocol violation that compromises the scientific rigor as determined by the data safety monitoring board, data will not be included in the analysis per the board recommendations. If data is missing, it will not be included in the analysis.

### Plans to give access to the full protocol, participant-level data and statistical code {31c}

The de-identified datasets for the current study can be made available by contacting the corresponding author upon reasonable request and in agreement with the local ethics committee.

## Oversight and monitoring

### Composition of the coordinating center and trial steering committee {5d}

This is a multicenter trial at the Veteran’s Affairs Hospitals in Cincinnati, OH, Providence, RI, and Boston, MA. Each site will have a Principal Investigator and study staff including research nurses/coordinators, research assistants, and pharmacy staff. The study staff will report to the PIs who will meet periodically with each other and study staff to ensure optimal progression of the trial. The Boston VA site is the study coordinating center that housed one senior research study coordinator for all three sites and will monitor the accurate conduction of the study at all sites and coordinates the monthly study coordinator meetings as well as the monthly trial steering committee meetings to ensure accurate execution of the study protocol. Providence VA is the data management center where all study data collection forms are produced and entered data reviewed for accuracy against source documents. The Cincinnati VA is the main funding site in charge of regulatory documents and study contracts. The trial steering committee is composed of the three PIs from the Cincinnati (JR), Boston (JJ), and Providence sites (WCW).

### Composition of the data monitoring committee, its role, and reporting structure {21a}

A central data monitoring committee organized and coordinated by Veterans Affairs, comprised of field experienced researchers, independent of the study investigators or the funding agency, will meet periodically (6 months to 1 year) to review adequate recruitment and conduction of the study including adverse events and protocol violations and will determine whether or not it is safe to continue the trial as described in detail in the Official Charter for the Study by the Clinical Science Research and Development office of the Department of Veterans Affairs. In addition, there is a central Institutional Review Board, independent of the DMC, the funding agency, and the investigators, who are responsible of the safety of the participants and ensuring the safe conduction of the study per protocol.

### Adverse event reporting and harms {22}

Any and all adverse events will be documented in the participant’s study records. Adverse events will be collected by study staff, relayed to the principal investigators, and reported to the data safety committee and Central IRB.

### Frequency and plans for auditing trial conduct {23}

The Central IRB, the funding agency (VA ORD), and the DMC have the authority to audit study charts and conduction at any point in time. In addition, to ensure our own data integrity, we will perform a blinded interim analysis at 50% of the recruitment target. The study will proceed as planned unless the difference in the absolute rate of adverse events between the two study arms are greater 20%, at which case the study will be halted and a balance of the risk vs benefit ratio of completing the study will be discussed with the Data Safety Monitory Board. We will perform blinded sample size re-estimation [22] during our interim analysis.

### Plans for communicating important protocol amendments to relevant parties (e.g., trial participants, ethical committees) {25}

Protocol amendments will be made at the discretion of the principal investigators or recommended by the Central IRB or DMC. Once approved by the ethical committees, study staff will be trained and the amendments will be implemented at the next study visit with the participants.

### Dissemination plans {31a}

The investigators plan to publish the results of this trial in a peer-reviewed journal. They may present a portion of this data at research conferences.

## Discussion

The Re-Prosper-HF study is the first multicenter, randomized clinical trial to test the efficacy of probenecid, a non-cAMP calcitrope, to improve cardiac function, function, and health status in HFrEF. If successful, the results of this trial will be used as data to the design of a large, multi-centered RCT, powered for clinical outcomes.

The results of this study will be particularly important to the field of inotropic study, as recent studies with related compounds have not yielded practice-changing results yet. The most recent trial with a non-cAMP inotrope was a phase II study that demonstrated that istaroxime infusion over 24 h improved systolic (measured by indexed stroke volume, ejection fraction was not significantly improved) and diastolic function but did not significantly improve BNP or symptomatology in comparison to placebo [6]. Similarly, a larger study using the myotrope omecamtiv mecarbil (the COSMIC-HF study) recently found a modest symptomatic improvement with short term therapy as measured by one methodology but no significant change as measured by a second (KCCQ) [23], while the larger GALACTIC-HF study found no significant difference in the treatment group in symptomatology or cardiovascular death but did report a small but statistically significant improvement in the composite endpoint [24].

These studies, as well as the smaller clinical studies with probenecid, demonstrate a clear potential for the development of novel inotropic therapies within the non-cAMP calcitropic and the myotropic pathways, but also demonstrate the significant difficulties in developing novel compounds. The ReProsper-HF study will build upon this acquired knowledge with a repurposed compound with a known safety profile after decades of clinic use. The results of this trial may provide not only a new medical option in the growing armamentarium for HFrEF, but may also provide proof of concept for non-cAMP-dependent calcitropes in improving cardiac function through a novel TRPV2 channel-dependent pathway.

## Trial Status

The Re-Prosper-HF study is on its first version of the protocol and is expected to begin enrollment on June 1, 2021. Completion of the study protocol is expected to be near May 30, 2025, and the estimated end of study date will be November 3, 2025.

## Abbreviations

HFrEF: Heart failure with reduced ejection fraction
cAMP: Cyclic adenosine monophosphate
HF: Heart failure
TRPV2: Transient receptor potential vanilloid type 2
CICR: Calcium-induced calcium release
LVEF: Left ventricular ejection fraction
ADHF: Acute decompensated heart failure
BEST: Bicycle exercise stress test
METS: Metabolic equivalents
KCCS: Kansas City cardiomyopathy questionnaire
